# Estro-progestin and progestogen intake: What’s the impact on hysteroscopic imaging?

**DOI:** 10.52054/FVVO.15.1.056

**Published:** 2023-03-31

**Authors:** G Garuti, M Colonnelli, A Soliani, C Lubrano, M Soligo

**Affiliations:** Department of Obstetrics and Gynaecology, Public Hospital of Lodi via Savoia, Lodi, Italy

**Keywords:** Hysteroscopy, Endometrium, Estro-progestins, Progestogens, Hormonal Replacement Therapy, Endometrial hyperplasia

## Abstract

**Background:**

In current literature there is no report aimed to evaluate the effects of exogenous steroids on hysteroscopic imaging.

**Objectives:**

To evaluate the hysteroscopic features of endometrium in women undergoing female hormones administration.

**Materials and Methods:**

We reviewed video-records of hysteroscopies carried-out in women taking estro-progestins (EP), progestogen (P) and Hormonal Replacement Therapy (HRT). All women underwent biopsies resulting in atrophic, functional, or dysfunctional pathological reports.

**Main Outcome Measures:**

Description of hysteroscopic pictures related to each schedule of therapy.

**Results:**

The study included 117 women. We evaluated 82, 24 and 11 women treated by EP, P and HRT, respectively. In EP users, imaging indistinguishable from physiological pictures was found when high oestrogen dosage and low-potency progestogen as 17-OH progesterone derivatives were administered. By enhancing progestogen potency with 19-norprogesterone and 19-nortestosterone derivatives we observed a promotion of progestogen differentiation such as polypoid-papillary pseudo-decidualisation, spiral artery differentiation, inhibition of gland-proliferation and endometrial atrophy. In P users we distinguished two patterns, depending on continuous or sequential schedules. Continuous therapy resulted in atrophic or proliferative-secretory features whereas sequential ones led to endometrial overgrowth reflecting stromal pseudo-decidualisation. Women undergoing HRT showed atrophic features in combined continuous and polypoid overgrowth in sequential schedules. In women taking Tibolone we found pictures ranging from atrophic to hyperplastic appearances.

**Conclusions:**

Exogenous steroids lead to significant endometrial moulding. Depending on schedule, hysteroscopic- view appears predictable and often showing overgrowths mimicking proliferative pathologies. In this case biopsy is recommended but in common practice physicians should gain awareness with hysteroscopic pictures induced from hormone administration.

**What is new?:**

Systematic assessment of hysteroscopic pictures during estro-progestins intake.

## Introduction

Hysteroscopy is currently considered the gold standard to assess endometrial pathology. Throughout the last two decades hysteroscopy was found to be a reliable diagnostic tool in predicting endometrial abnormalities such as polyps, submucous myomas, endometritis, hyperplasia, and carcinomas, showing various degree of accuracy (Garuti et al., 2001; Garuti et al., 2006; Dueholm et al., 2015; Di Spiezio Sardo et al., 2022). The endometrium is the most dynamic tissue of the human body, its moulding and appearance depending on tissue-specific biochemical pathways activated by endogenous or exogenous steroids hormones binding to families of Oestrogens (ER α and β) and Progesterone (Pg R A and B) receptors, that are highly represented in both epithelial and stromal endometrial cells (Hewitt et al., 2016; Wagenfeld et al., 2016). While the hysteroscopic-view of physiological and pathological endometrium has been widely described, there are currently no literary reports aimed at assessing the effects of female hormone intake on the endometrial imaging (Garuti et al., 2001; Garuti et al., 2005). The scope of the present study is to evaluate the features of hysteroscopic- view in healthy women treated currently by various schedules of Estro-Progestins (EP) and Progestogens (P) drugs.

## Patients and methods

This observational study is based on the review of hysteroscopic examinations accomplished throughout a 5-year period (from January 2016 to December 2021) by a single surgeon (GG), collecting video recorded clips of women currently treated by EP and P, referred to the public Hospital of Lodi (Italy). The drugs were administered for either contraceptive requirements or to treat functional uterine bleeding in pre-menopausal and as Hormone Replacement Therapy (HRT) in menopausal women. The patients underwent hysteroscopic assessment because of abnormal uterine bleeding and/or abnormal endometrial thickness or eco-texture, based on transvaginal ultrasound findings. Pathological reports were retrieved by a search from our Institutional database. The criteria for patient inclusion were the availability of good-quality video clips and the accomplishment of an endometrial biopsy resulting in functional, dysfunctional, and atrophic endometrium (Buckley and Fox, 1989). Depending on patient preference and clinical background, hysteroscopy was performed as either an outpatient clinic or in surgical room under conscious sedation. All patients underwent hysteroscopy with 14Fr to 16Fr-sheathed hysteroscopes tipped with a 30° lens and a 5Fr-working channel. Normal saline was used as liquid distension medium and it was delivered by an irrigating-suction device. Endometrial sampling was obtained under hysteroscopic guidance by 5Fr-mechanical instrumentation, addressing the biopsy to focal lesions or randomly in cases of evenly lined endometrium. Based on endometrial morphology, we classified hysteroscopic imaging as follows: (Garuti et al., 2001; Garuti et al., 2005; Dueholm et al., 2015).

### Atrophic endometrium

This condition describes a thin, smooth mucosa evenly shaping the endometrial cavity showing few gland openings and cases in which no gland openings were found while a very thin endometrium mirrored the trabecular pattern of underlying myometrium.

### Functional endometrium

These women displayed both proliferative and secretory visual pathways. Proliferative features were characterised by a smooth endometrial surface or minimal roughness in an evenly lined uterine cavity, showing a regular arrangement of gland openings. Secretory endometrium identified a thicker evenly lined mucosa, with a smooth and velvety surface maintaining regularly-spaced gland openings.

### Polypoid endometrium

This picture displayed an uneven and thick endometrium, showing velvety, broad-based, or pedunculated focal mucosal projections.

### Papillary endometrium

This picture presented a velvety and evenly shaped mucosa showing small projections (1 to 3 mm).

### Hyperplastic endometrium

Describing a thickened endometrium with rough, uneven surface showing polypoid projections with gland-cysts and irregularly spaced or crowded gland openings.

### Mixed features

Co-existence of two or more of previously described pictures.

## Results

We identified 117 women satisfying the inclusion criteria. In Table I we show the schedules of treatment and the number of women who underwent hysteroscopic assessment in each of them. Combined EP, only P either continuous or sequential and HRT were evaluated in 82, 24 and 11 women, respectively. In combined EP schedules, progestogens were represented from four molecular classes: 1. 17-OH Progesterone derivative (Cyproterone and Clormadinone), 2. 19-Norprogesterone derivative (Nomegestrole), 3. 19-Nortestosterone derivative (Desogestrel, Etonogestrel, Gestodene, Norelgestromin, Levonorgestrel, Norgestimate and Dienogest), 4. Spironolactone derivative (Drospirenone) whereas oestrogens were included either as ethinyl-oestradiol (EE) or natural oestrogens such as oestradiol 17β or oestradiol-valerate. Only progestogen administration was based either as continuous or sequential administration of Progesterone, Desogestrel, Dienogest, Etonogestrel, Medroxyprogesterone, Norethisterone and Megestrol. Continuous or sequential HRT schedules were administered as oestradiol-17β cutaneous patch, Tibolone and Dihydrogesterone, Drospirenone, Cyproterone combinations with oestradiol-17β or oestradiol-valerate. We then describe hysteroscopic imaging associated with the administration of these categories of drugs and schedules.

**Table I:** Schedules of drugs currently administered to the 117 patients undergoing hysteroscopic assessment.

Drug schedule	Number of women
Cyproterone acetate2 mg / EE 35 mcg	4
Clormadinone acetate 2 mg/EE 30 mcg	3
Nomegestrole acetate 2.5 mg/ oestradiol-17β 1.5 mg	18
Drospirenone 3 mg / EE 20 - 30 mcg	6
Desogestrel 25-150 mcg /EE 20 - 40 mcg	9
Ethonogestrel 120mcg/day // EE 15mcg/day (vaginal ring)	2
Gestodene 60 -75 mcg/ EE 15 - 40 mcg	11
Levonorgestrel 100 mcg / EE 20 mcg	7
Dienogest / oestradiol valerate (quadriphasic combination) and Dienogest 2mg/30 mcg EE	15
Norgestimate 250 mcg /EE 35 mcg	1
Norelgestromin 150 mcg/day // EE 20mcg/day (cutaneous patch)	6
Progestogen only (continuous and sequential)	24
Hormone Replacement Therapy (continuous combined and combined sequential, tibolone, oestrogens)	11
Total	117

### Estro-progestins

#### 17-OH progesterone derivatives

**Figure 1:**
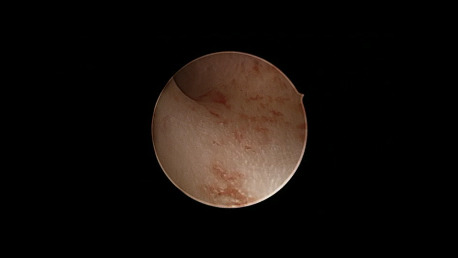
Cyproterone acetate 2mg/EE35mcg. An evenly lined mucosa showing regular architecture of gland opening reproduces endometrial middle proliferative features.

Cyproterone acetate 2 mg / EE 35 mcg (4 women): In all women we found a functional picture indistinguishable from proliferative endometrium (Figure 1).

Clormadinone acetate 2 mg / EE 30 mcg (3 women): Functional secretory appearances were shared from all examined patients.

#### 19-Norprogesterone derivative

**Figure 2:**
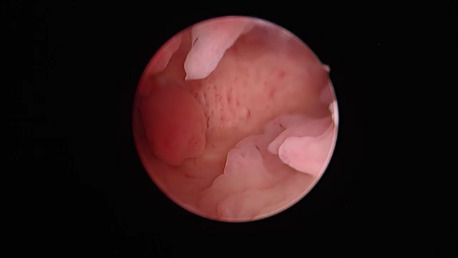
Nomegestrole acetate 2.5 mg/oestradiol-17β 1.5 mg. Uneven endometrial lining showing marked polypoid differentiation mixed with atrophic appearances.

Nomegestrole acetate 2.5 mg/oestradiol 17β 1.5mg (18 women): We observed different and sometimes mixed hysteroscopic patterns. Atrophic, polypoid, papillary, secretory, and mixed features were found in 5, 3, 3, 1 and 6 women, respectively (Figure 2).

#### 19-Nortestosterone derivatives

Desogestrel 25-150 mcg and EE 20-40 mcg (9 women): In 8 women undergoing monophasic schedule based on Desogestrel 150 mcg and EE 20-30 mcg, we found a background of atrophy associated with scattered papillary or polypoid features. In 1 woman undergoing biphasic combination characterised by less progestogen (25-150 mcg) and higher estrogenic (30-40 mcg) milieu, we found a proliferative pattern.

Etonogestrel 120 mcg and EE 15 mcg (vaginal ring, 2 women): Atrophy mixed with focal papillary and polypoid overgrowth were found in both women. Gestodene 60-75 mcg and EE 15-40 mcg (11 women): In 10 women taking monophasic combined schedules we found endometrial atrophic, secretory, and papillary pattern in 3, 3 and 4 cases, respectively. In 2 of these women the vascular network showed focal or extensive spiral artery differentiation. In one woman taking a triphasic combination (Gestodene 50-70-100 mcg/EE 30-40-30 mcg) an atrophic picture was observed.

**Figure 3:**
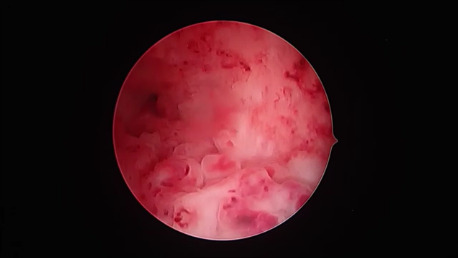
Levonorgestrel 100mcg/EE 20mcg. Homogeneous papillary overgrowth showing coiled vessels differentiation.

Levonorgestrel 100 mcg and EE20 mcg (7 women): We detected an atrophic picture in 4 women and in one case an atrophic background was mixed with scattered papillary projections. In two women we found even papillary features with associated spiral artery differentiation (Figure 3).

Dienogest 2 mg and EE30 mcg (5 women), Dienogest 2-3 mg and oestradiol valerate 1-3 mg (10 women): The monophasic combination of Dienogest with EE yielded inspective findings of atrophy and proliferative features in 2 and 3 women, respectively. The quadriphasic schedule associating Dienogest with oestradiol valerate showed atrophy, secretory, proliferative and polypoid features in 5, 3, 1 and 1 women, respectively.

Norgestimate 250 mcg and EE 35 mcg (1 woman): A functional secretory endometrial picture was found in only one assessed woman.

Norelgestromin 150 mcg and EE 20 mcg (cutaneous patch, 6 women): Proliferative and atrophic pictures were observed in 4 and 2 women, respectively.

#### Spironolactone derivative

Drospirenone 3mg and EE 20-30mcg (6 women): We found an atrophic endometrium in 3 out of 6 women whereas in 1 and 2 cases we detected secretory and mixed findings sharing proliferative and papillary features.

#### Progestogens

Continuous schedules: We evaluated 17 women undergoing continuous administration of Desogestrel 75 mcg (8 women), Dienogest 2 mg (3 women), Etonogestrel (68 mg subcutaneous implant, 2 women), Norethisterone 2.5 mg and 10 mg (3 woman), Megestrol 160 mg (1 woman).

Desogestrel 75 mcg: Evenly lined endometrium showing atrophy and papillary features was found in 4 and 1 woman, respectively. Mixed pictures of atrophy-proliferative, papillary-proliferative, and papillary-polypoid features were found in the remaining 3 women.

Dienogest, 3 mg: Atrophy and mixed pictures of atrophy with papillary and proliferative features were observed in 1, 1 and 1 women, respectively.

Etonogestrel (subcutaneous implant): The assessment of 2 women showed atrophy in one case and a mixed picture of atrophy with papillary differentiation in the other.

Norethisterone acetate, 2.5 mg and 10 mg: In one woman tacking 2.5 mg atrophic features were observed whereas in two cases treated by 10 mg we found papillary differentiation.

Megestrol acetate (160 mg): An atrophic picture was found in the only examined woman.

**Figure 4:**
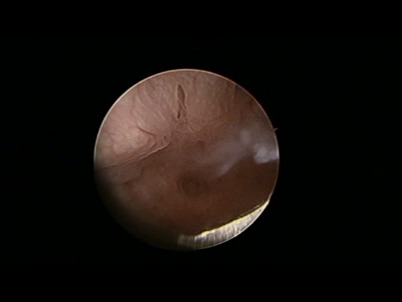
Sequential 10 mg Norethisterone-acetate administration displaying polypoid features with marked secretory differentiation.

Sequential schedules: We assessed 7 women treated by P administered as sequential schedule, throughout a 12–15-day period during the secretory phase of the menstrual cycle. The administered drugs were Progesterone 100 mg, Dihydrogesterone 10 mg, Medroxyprogesterone acetate 10 mg, Nomegestrol acetate 5 mg and Norethisterone acetate 10 mg (3 patients). All women underwent hysteroscopy during P intake. In all cases we found an even polypoid overgrowth associated with secretory features (Figure 4).

#### Replacement Therapy

We reviewed hysteroscopy of 11 women undergoing HRT. Among these unopposed oestrogens, continuous combined and sequential combined schedules were administered to 1, 3 and 2 women, respectively. Moreover, we evaluated 5 women taking Tibolone.

**Figure 5:**
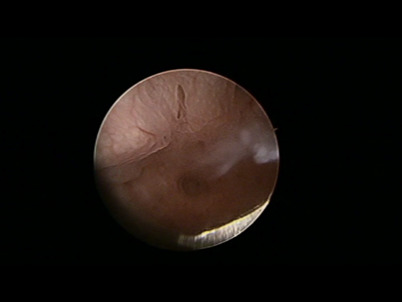
Oestradiol cutaneous patch, 25 mcg/day. In the only woman treated by unopposed oestrogens, we found a rough polypoid overgrowth with irregular gland architecture and gland-cyst differentiation, a picture indistinguishable from endometrial hyperplasia.

Unopposed ERT. Oestradiol cutaneous patch 25mgc/day: Hysteroscopic-view was characterised by mixed polypoid and hyperplastic features in only one woman undergoing a schedule as such (Figure 5).

Combined continuous HRT. Oestradiol 17-β 1mg/Drospirenone 2 mg: In the 3 women evaluated, we observed focal mucosal thickening in a background of an atrophic endometrium.

**Figure 6:**
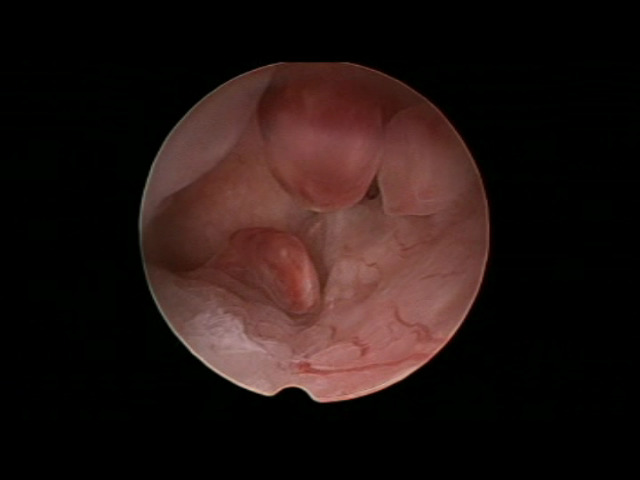
Oestradiol 17-β 1 mg, Dihydrogesterone 10 mg. Uneven polypoid and papillary endometrial overgrowth with scattered gland cyst formation cannot be distinguished from hyperplasia.

Combined sequential HRT. Oestradiol valerate 2 mg/Cyproterone acetate 1 mg (1 woman), oestradiol 17-β 1 mg/Dihydrogesterone 10 mg (1 woman): We observed a combination of polypoid and hyperplastic features in both women evaluated during the window of progestogen administration (Figure 6).

**Figure 7:**
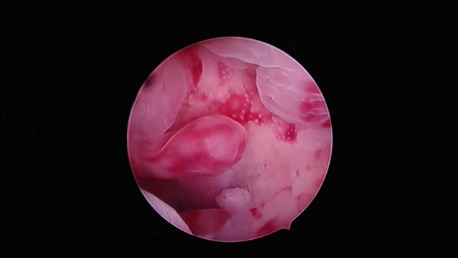
Tibolone 2.5 mg. An even polypoid endometrial growth mixed with focal crowding of gland openings and enhanced vascular network are suggestive of endometrial hyperplasia.

Tibolone 2.5 mg: We found different pictures in the 5 women evaluated. In 2 women we observed an atrophic endometrium whereas mixed hyperplastic and polypoid features were found in the other 3 cases (Figure 7).

The summary of hysteroscopic-view findings is shown in Table II. As reported in the Methods section, all patients underwent one or more hysteroscopy-targeted mechanical biopsy. In no case was the pathologic report consistent with polyp, hyperplasia, or carcinoma. In all cases displaying an unevenly lined endometrium (polypoid, papillary, and hyperplastic) stromal pseudo-decidualisation and secretory features were found at pathologic assessment whereas atrophic endometrium, proliferative and dysfunctional histopathologic reports were obtained in the other cases.

**Table II:** Summary of hysteroscopic-view findings obtained in 117 patients undergoing estro-progestins and progestogens administration. Abnormal polypoid and papillary features are put together in a same class.

	Estro-progestin	Continuous progestogen	Sequential progestogen	ERT/HRT/Tibolone	
Hysteroscopic view					Total
Atrophic endometrium	25 (30.4%)	8 (47.0%)		2 (18.1%)	35 (29.9%)
Functional endometrium	29 (35.3%)	1 (5.8%)			30 (25.4%)
Polypoid/Papillary endometrium	9 (10.9%)	3 (17.6%)	7 (100%)		18 (15.3%)
Mixed features	19 (23.1%)	5 (29.4%)		9 (81.8%)	33 (28.2%)
Total	82	17	7	11	117

Six cases showing hyperplastic features have been included in the mixed features class and all of them fell in the group undergoing menopausal Hormone Replacement Therapy.

*(ERT: Estrogen Replacement Therapy, HRT: Hormone Replacement Therapy).*

## Discussion

The exposure of endometrium to exogenous sex female hormones leads to a variety of known dysfunctional histopathologic pictures, varying with respect to the administered schedule of treatment (Buckley and Fox, 1989). Our knowledge about the mechanism of action of oestrogens and progestins have evolved from the concept of nuclear receptor-mediated regulation of transcription to a sophisticated interplay between autocrine and paracrine coregulators and signalling cascades, resulting in the specific morphological results on target tissues (Hewitt et al., 2016; Wagenfeld et al., 2016; Eyster, 2016). Many pathophysiological and pharmacologic variables can affect the endometrial moulding during female steroid administration. Among these, individual extra-gonadic endogenous oestrogen production, Sex Hormone Binding Globulin plasma levels, time, route and schedule of administration and the different biological potency of oestrogens and progestogens may be of concern in determining variable morphologic patterns of endometrium (Bick et al., 2021). Hysteroscopy assessment of endometrial lining, with or without targeted endometrial biopsy, is the current gold standard diagnostic to carry out endometrial pathologies. Nevertheless, in current literature there is no report aimed at establishing the relationship between female steroid intake and hysteroscopic imaging.

In the 82 women undergoing EP treatments the biological potency of progestogen, the dosage of EE and the inclusion of natural oestrogens in the drug combination led to different hysteroscopic pictures. Low dosages of weak 17-OH progesterone derivatives such as Cyproterone and Clormadinone, when combined to 30 to 35 mcg of EE resulted in the maintenance of a regular gland architecture and an endometrial lining indistinguishable from physiological proliferative or secretory endometrium in all examined patients. The shifting to schedules containing more potent P, such as 19-norprogesterone and 19-nortestosterone derivatives, mainly when lowering the dosage of EE to 15-20 mcg or including a weaker oestrogen such as oestradiol-17β or oestradiol-valerate led to different hysteroscopic pictures. Endometrial atrophy, polypoid and papillary patterns, paucity of gland network and spiral artery differentiation were mainly found in this group of women, to express the prevalence of progestogen biochemical effects by inhibition of the oestrogen pathways and the promotion of endometrial stromal pseudo- decidualisation. Similarly, drospirenone combined with either 20 and 30 mcg of EE showed both atrophic and papillary hysteroscopic patterns, consistent with a highly effective progestogen action. Nevertheless, proliferative, and secretory features were often observed in women taking schedules based on higher oestrogen dosage, such as monophasic 30 mcg EE/Dienogest, 35 mgc EE/Norgestimate, biphasic 30-40 mcg EE/ desogestrel and in patients treated by transdermal EE/Norelgestromin, suggesting that these combinations supply an oestrogenic milieu leading to either a persistent proliferative moulding or secretory differentiation indistinguishable from physiological pictures.

The administration of P only resulted in two substantially different hysteroscopic patterns. In women undergoing continuous treatments we found a prevalence of atrophic features sometimes combined with papillary or proliferative pictures. In all patients in whom hysteroscopy was scheduled during sequential progestogen intake, we observed an endometrial overgrowth showing polypoid appearances with secretory features, to express a significant endometrial pseudo-decidualisation. This overgrowing decidual differentiation, mimicking endometrial hyperplasia, reflects the widespread prior oestrogen-mediated induction of progesterone receptors.

In the menopausal women undergoing HRT we also observed different hysteroscopic pictures based on the administration of continuous or sequential schedules. Continuous EP administration containing a weak oestrogen resulted in substantially atrophic features whereas in all women taking only oestrogen or sequential EP formulations we found polypoid and hyperplastic features. These findings are consistent both with persistent proliferative stimulation determined by a therapy based only on oestrogen administration as well as throughout an oestrogen-mediated induction of progestogen receptors, leading to stromal decidualisation due to the subsequent progestogen effect. Tibolone, an analogue of the progestin norethynodrel, is largely used against menopausal symptoms. Its activity is based on bioconversion to metabolites showing either oestrogenic and progestogenic properties (Kloosterboer, 2004). Tibolone intake led to different endometrial features, ranging from atrophy to significant gland stimulation and polypoid pseudo-decidualisation indistinguishable from endometrial hyperplasia. Individual differences in metabolic conversion to the active metabolites of the drug may be the cause of variable effects on endometrial imaging.

## Conclusions

The biological potency and dosage of both oestrogen and progestogen contained in EP schedules influence endometrial imaging, ranging from features indistinguishable from physiological proliferative or secretory phases to pictures of atrophy and stromal pseudo-decidualisation in a quite predictable manner. In both premenopausal and menopausal women, sequential P administration after oestrogen priming resulted in the hysteroscopic-view showing polypoid features related to stromal decidualisation. Endometrial overgrowth shared both by papillary and polypoid appearances has been frequently found in EP and P users and, mainly when associated with gland proliferation or vascular network prominence it can mirror hyperplasia or early endometrial cancer. Therefore, in common practice any significant endometrial thickening, even if expected because of current hormonal therapy, should undergo endometrial sampling for pathological assessment. Nevertheless, all physicians addressed or dedicated to the hysteroscopy field must gain awareness and familiarity with endometrial dysfunctional pictures induced from female steroid administration.
